# Generation Using Phage-Display of pH-Dependent Antibodies Against the Tumor-Associated Antigen AXL

**DOI:** 10.3390/antib14040083

**Published:** 2025-09-30

**Authors:** Tristan Mangeat, Célestine Mairaville, Myriam Chentouf, Madeline Neiveyans, Martine Pugnière, Giang Ngo, Vincent Denis, Corentin Catherine, Alexandre Pichard, Emmanuel Deshayes, Margaux Maurel, Matthieu Gracia, Anne Bigot, Vincent Mouly, Sébastien Estaran, Alain Chavanieu, Pierre Martineau, Bruno Robert

**Affiliations:** 1 Institut de Recherche en Cancérologie de Montpellier (IRCM), Université Montpellier, Institut Régional du Cancer de Montpellier (ICM), INSERM, 34298 Montpellier, France; tristan.mangeat@inserm.fr (T.M.); pierre.martineau@inserm.fr (P.M.); 2 Institut Régional du Cancer de Montpellier (ICM), Service de Médecine Nucléaire, 34298 Montpellier, France; 3 Institut de Myologie, Centre de Recherche en Myologie, Sorbonne Université, 75013 Paris, France; 4 Institut des Biomolécules Max Mousseron, Université Montpellier, Centre National de la Recherche Scientifique (CNRS), Ecole Nationale Supérieure de Chimie de Montpellier (ENSCM), 34090 Montpellier, France

**Keywords:** phage display, acidic pH, pH dependent antibodies, AXL, cancer immunotherapy, Warburg effect

## Abstract

Background/Objectives: Tumor-associated antigens are not tumor-specific antigens but proteins that are overexpressed by tumor cells and also weakly expressed at the surface of healthy tissues. Therefore, some side effects are observed when targeted by therapeutic antibodies, a phenomenon named “on-target, off-tumor toxicity”. As tumors generate an acidic microenvironment, we investigated whether we could generate pH-dependent antibodies to increase their tumor specificity. For this proof-of-concept study, we selected the tyrosine kinase receptor AXL because we already developed several antibodies against this target. Methods: To generate a pH-dependent anti-AXL antibody, we performed classical panning of a single-chain variable fragment (scFv) library using phage display at an acidic pH throughout the process. Results: After the third round of panning, 9 scFvs, among the 96 picked clones, bound to AXL at acidic pH and showed very low binding at a neutral pH. After reformatting them into IgG, two clones were selected for further study due to their strong pH-sensitive binding. Using molecular docking and alanine scanning, we found that their binding strongly depended on two histidine residues present on AXL at positions 61 and 116. Conclusions: To conclude, we set-up an easy process to generate pH-dependent antibodies that may increase their tumor-binding specificity and potentially decrease toxicity towards healthy tissues.

## 1. Introduction

Monoclonal antibodies have significantly improved the management of patients with cancer. As many antibodies without any modification (i.e., naked antibodies) have a limited therapeutic efficacy, several strategies have been developed to increase their anti-cancer activity. For instance, antibodies can be coupled with cytotoxic molecules to generate a new class of drugs called Antibody-Drug Conjugates (ADCs) [1], or they can be engineered to obtain bispecific antibodies that trigger the activation of CD3+ cells against tumor cells (i.e., T-cell engagers or TCEs) [2]. Moreover, T cells can be genetically modified to express at their surface antibody fragments in order to redirect them and target tumor cells. These chimeric antigen receptor-T (CAR-T) cells have showed impressive results in patients with blood malignancies [3], whereas the results are mitigated in solid tumors. However, these strategies also increase toxicity compared with naked therapeutic antibodies that show limited and easily manageable side effects. This is mainly due to the early release of the payload outside the tumor [4] or due to overstimulation of the immune system that becomes difficult to control in patients and that could even cause death [5].

Some side effects occur mainly because of the binding of these new immunotherapeutic agents to the target that is weakly expressed at the surface of healthy tissues, a phenomenon described as “on-target off-tumor toxicity”. Indeed, the vast majority of molecules targeted by therapeutic antibodies are not tumor-specific but overexpressed by tumor cells. Therefore, the therapeutic window of ADCs, T-cell engagers, and CAR-T cells could be increased by developing antibodies with higher binding capacity to their target in the tumor than in healthy tissues. By finely modulating their affinity/avidity and format, such antibodies will bind weakly and transiently to the antigen weakly expressed on normal cells but strongly to the same antigen highly expressed on cancer cells [6,7,8,9]. However, the avidity-based approach needs to be suited to the antigen according to its expression level. To overcome the need for customized design, another strategy is to generate a pro-antibody by masking its paratope with a universal coiled-coil masking domain that is then selectively removed by proteases present in the tumor microenvironment, thus avoiding binding of the pro-antibody to healthy tissue cells [10]. Another strategy is to exploit the different features of the tumor and healthy tissue microenvironments that may modulate antibody–antigen interactions. For example, in solid tumors, glycolysis and lactate production are increased, leading to proton accumulation and consequently to pH acidification of the microenvironment, from 7.2–7.4 in healthy tissues to 6–6.5 in tumors [11,12]. Here, we evaluated whether phage-display selection could be used for the fast and efficient development of pH-dependent antibodies that better bind to their targets at a slightly acidic pH. As proof of concept, we chose the tyrosine kinase receptor AXL as the target because it is implicated in resistance to chemotherapy and in the recruitment of immunomodulator macrophages at the tumor site [13]. This receptor is expressed on tumor cells but also on immune cells, such as natural killer cells [14], and cells in other healthy tissues (The human protein atlas). We hypothesized that a pH-dependent anti-AXL antibody would bind to its target predominantly on tumor cells and not on healthy tissue and non-tumor natural killer cells

## 2. Materials and Methods

### 2.1. Selection and Screening of pH-Dependent Single-Chain Variable Fragment (scFv) Clones

The previously described human scFv HuscI library [15,16] was used for phage display selection. Before phage selection on the AXL target, a depletion step of the naïve phage pool was performed on the recombinant dimeric human TYRO3 extracellular domain (ECD) fused to a human Fc and tagged with hexahistidine (TYRO3-ECD-Fc-6his Ref. 859 -DK, R&D Systems) and recombinant human IgG1-Fc (home-made) adsorbed on plastic plates (Nunc Maxisorp plate, ThermoFisher Scientific, Waltham, MA, USA) in PBS overnight at 4 °C and further saturated with bovine serum albumin (BSA). Soluble phages recovered from this depletion step were used to perform three rounds of selection on recombinant dimeric human AXL-ECD-Fc-6His (Ref. 154-AL, R&D Systems—Bio-Techne, Minneapolis, MN, USA) adsorbed on plastic plates. BSA and milk were used alternately to sature plastic plates. All described steps were performed in citrate buffer at pH 5.5. Recovery of the binder phage was performed after trypsin digestion and used to infect TG1 bacteria.

The 96 soluble scFv clones produced by secretion from HB2151 cells (*Escherichia coli* strain) were screened in two parallel ELISAs performed in citrate-phosphate buffer at pH 5.4 and phosphate-buffered saline (PBS) at pH 7.4, using AXL-ECD-Fc-6His and TYRO3-ECD-Fc-6His as target proteins. Binding of the scFv clones was detected using an HRP-coupled anti-MYC antibody (clone 9E10, Santa Cruz Biotechnology, Dallas, TX, USA) and revealed by adding the TMB substrate and stopping the reaction with 1M H_2_SO_4_. Plates were read at 450 nm.

### 2.2. Production of Recombinant pH-Dependent IgG Clones and Their Validation

Positive scFv clones (i.e., clones that bound to AXL at acidic pH) were cloned as fully human IgG1 in the pEF vector after PCR amplification or using synthetic fragments (Twist Biosciences, San Francisco, CA, USA) in which potential N-glycosylation sites (NXS/T consensus sequence) were removed using Golden Gate. Recombinant antibodies were generated by transient transfection of HEK293T cells. Plasmid-encoding IgG was complexed with polyethyleneimine (PEI) (Polysciences, Warrington, PA, USA) for 15 min in 150 mM NaCl and then deposited on HEK293T cells. After 4 h of incubation at 37 °C, the medium was removed, and cells were incubated in serum-free medium. After 6 days, the supernatant was collected and centrifuged to remove cells and debris. Recombinant IgG was captured from the clarified supernatant using protein A-agarose overnight under agitation at 4 °C. The protein A-agarose-filled column was then washed with 10 volumes of PBS, and IgG was eluted with 0.2 M glycine buffer at pH 2.7 and then quickly buffered with 1 M Tris at pH 9. The collected monoclonal antibodies are quickly transferred to PBS using PD-10 desalting columns containing Sephadex G-25 resin (Cytiva, Malborough, MA, USA). IgG quantification was performed at 280 nm using an extinction coefficient of 1.3 mL mg^−1^ cm^−1^, and integrity was confirmed by SDS-PAGE. High-affinity anti-AXL antibody Ig_HA was generated using VH and VL sequences from the WO2017180842 patent. Binding of the recombinant human IgG1 clones to AXL was validated by ELISA using AXL-ECD-Fc-6His, followed by detection with peroxidase-conjugated goat anti-human Fab (A0293, Sigma-Aldrich, Saint Louis, MO, USA).

### 2.3. Cell Lines and Flow Cytometry

The MDA-MB-231, HEK293, and HEK293T cell lines from ATCC were cultured as recommended. Immortalized human myoblasts (AB1190, AB1079, KM670, AB1167, and AB678) [17] were grown in Skeletal Muscle Cell Growth Medium (C-23060, PromoCell, Heidelberg, Germany) with 20% fetal bovine serum under 5% CO_2_ at 37 °C.

For flow cytometry, 5 × 10^5^ cells in PBS supplemented with 3% BSA at pH 6.0 or pH 7.4 were incubated with the primary antibody on ice for 1 h. After washes with the corresponding buffer, cells were incubated with A647-labeled polyclonal goat anti-human Fab (AB_2337881, 109-605-006, Jackson ImmunoResearch, Ely, UK) in the corresponding buffer on ice for 1 h. Finally, after three washes with the corresponding buffer, labeled cells were visualized using the CytoFLEX platform (Beckman Coulter, Brea, CA, USA), and results were analyzed with FlowJo.

### 2.4. Production of Recombinant AXL Domains

Synthetic codon-optimized sequences of AXL Ig domains 1 and 2 (1–227), Ig domain 1 (1–138), and mutated Ig domain 1 (H61A, H116A, and H61A plus H116A) (Twist Biosciences, San Francisco, CA, USA) were cloned in frame with human IgG1 Fc-6His by Golden Gate. After sequencing, positive plasmid constructs were used to produce the recombinant proteins by transient transfection in HEK293T cells with PEI (Polysciences, Warrington, PA, USA). After 6 days of incubation, supernatants were collected, and the recombinant proteins were purified as described previously for antibody purification. The purity of the recombinant proteins was verified by SDS-PAGE. The recombinant full-length AXL-ECD-Fc chimera was purchased from R&D Biotech.

### 2.5. Expression of AXL Domains at the Surface of HEK293-Fc-TM Cells

A stable doxycycline-inducible HEK293 cell line was generated by lentiviral infection of a construct that contains the TetOn operator followed by the sequences encoding the human IgG1 Fc domain with connecting plus transmembrane region (i.e., HEK293-Fc-TM line). In the presence of doxycycline, a high number of membrane-linked Fc domains were detected by flow cytometry using a FITC-labeled murine anti-human Fc monoclonal antibody (CSA 3236; Cohesion Biosciences, London, UK). Transient transfection of this cell line with the cDNAs encoding the different AXL extracellular domains fused to human IgG1 Fc-6His, PEI, and doxycycline generated, after 36 h, a cell population with monomeric AXL domains attached to the cell membrane by a heterodimeric Fc domain (one chain with the transmembrane domain and the other one without). The 6His tag was detected using a mouse anti-polyhistidine monoclonal antibody (Clone # AD1.1.10R, MAB050, R&D), followed by A488-labeled goat anti-murine Fab (AB_2536161, A28175, ThermoFisher Scientific, Waltham, MA, USA).

### 2.6. Surface Plasmon Resonance (SPR) Analysis

SPR analysis was performed with BIAcore and human AXL-ECD-Fc (R&D Systems- Bio-Techne, Minneapolis, MN, USA) covalently immobilized on a CM5 chip at pH 4 (Amine coupling kit, Cytiva). In this kinetic protocol, the equilibrium dissociation constants (KD) were determined in a single cycle by successive injections of the analyte at increasing concentrations separated by short dissociation times between injections. To determine the dissociation rate constant (koff), the dissociation time after injection of the last concentration was increased. Specifically, antibodies were injected at five increasing concentrations (2:2 dilution in PBS at pH 6.5 or pH 7.5; G10aPh: 0.17–0.35–0.70–1.4–2.8 nM; D4apH: 0.087–0.17–0.35–0.70–1.4 nM; and IgG-Ref: 0.087–0.17–0.35–0.70–1.4 nM). The double blank was subtracted from the response curves: one with an injection of buffer and another with a blank flow cell (with the same chemistry coupling but without protein). The corrected curves were directly given by the T200 evaluation software. The data were fitted to a model taking into account bivalent binding to determine the affinities of the antibodies.

### 2.7. Antibody-Antigen Docking and Analysis of the Binding Mode

The crystal structure at the 3.3 Å resolution of the human GAS6-AXL complex with a 2:2 stoichiometry was obtained from the Protein Data Bank (PDB ID: 2C5D) [18]. This structure comprises a dimer of a complex that involves immunoglobulin-like domains1 and 2 (Ig1 and Ig2) of the AXL ectodomain interacting with the first laminin G-like domain of GAS6. Before docking, the protein complex was prepared at pH 6 using the preparation module of BIOVIA Discovery Studio 2020 software and atom-typed with the CHARMM-36 force field tool (BIOVIA, Dassault Systèmes, Vélizy-Villacoublay, France). The scFv structure models of D4apH and G10apH were generated with Modeler [19] using the top 5 template approach in the Antibody Modeling Cascade integrated in Discovery Studio. The reference structure for the Complementarity-Determining Region (CDR) loops was the anti-gankyrin scFv structure (PDB ID: 4NIK) from the PMEW library [20]. Consistent with the MODELLER protocol, the loop refinement process was applied to all CDRs [21].

Both scFv structures were prepared at pH 6 and atom-typed before docking. Using the CDRs as the ligand site, each scFv structure was docked onto a specific region of the AXL monomer in complex with GAS6. This region encompassed the Ig1 domain and the start of the Ig2 domain. A total of 2000 poses were generated through rigid-body docking using ZDOCK. Only poses where at least one atom of the scFv was within a 15 Å radius sphere centered on AXL histidine residues 61 and 116 were selected. The selected poses were then refined using the RDOCK algorithm [22] and clustered with an RMSD cutoff of 5 Å.

For the most populated cluster, the binding modes of the 91 poses of D4apH and the 134 poses of G10apH were analyzed to identify potential interactions with residues His61 and His116 of AXL. This analysis was performed using the relaxed mode of the Residue Interaction Network Generator on both clusters [23] to identify antibody residues that, within the docking poses, are likely to engage in non-covalent interactions with the imidazole groups of the histidine residues. As no extensive minimization of side chains was performed before the RING analysis, the non-covalent interactions and their frequencies should be interpreted as indicative of potential interactions within each cluster, applying a cutoff frequency of 15%. Nevertheless, this approach suggests antibody residues that are likely involved.

## 3. Results

### 3.1. Selection of pH-Dependent Anti-Human AXL scFv Clones

**Figure 1:**
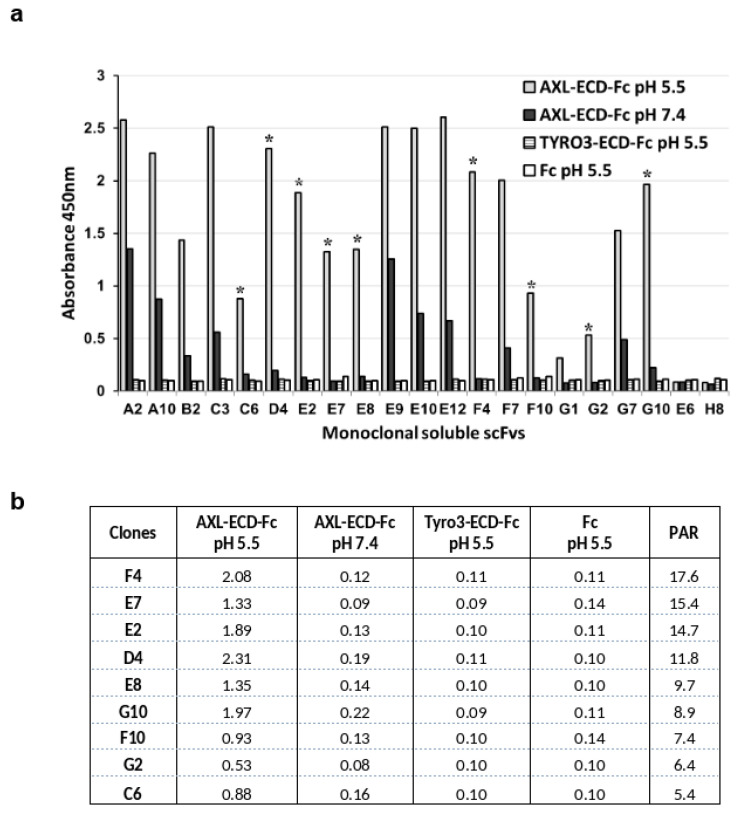
The identified scFv clones against human AXL display pH-dependent binding (i.e., low binding at physiological pH and high binding at acidic pH). After three rounds of panning of a phage scFv library, the pH-dependent binding properties of secreted scFv clones were analyzed by ELISA. (**a**) scFv clones were added to plates coated with recombinant AXL-ECD-Fc at pH 7.4 (black bar) and pH 5.5 (gray bar). The binding specificity was assessed by coating plates with human TYRO3-ECD-Fc (dotted bar) or human Fc (white bar) at pH 5.5. (**b**) The pH-dependent binding properties of the identified scFv clones were compared by calculating their pH-dependent binding activity ratio (PAR) as follows: signal ratio at pH 5.5/signal ratio at pH 7.4 (signal measured by ELISA). Clones with a PAR > 5 are indicated with a star in Panel (**a**).

To force the selection of scFv clones that bind to the AXL extracellular domain at acidic pH, we performed all the phage display selection steps at pH 5.5 after confirming that (i) ELISA with AXL-ECD-Fc-6His (coating and incubation) could be performed at this pH, and (ii) phages incubated at this pH could infect *E. coli* cells. Before panning, we carried out a depletion step using recombinant human TYRO3-ECD-Fc-6His as the antigen to avoid selecting phages against the Fc-His domain of the recombinant antigen and the homologous TYRO3 protein. Then, we performed three rounds of panning using AXL-ECD-Fc-6His (coated on plastic) and our proprietary naive human scFv synthetic library displayed on filamentous phages (HuscI library) at pH 5.5 in citrate-phosphate buffer. Analysis of the phage-scFv pool by ELISA confirmed enrichment for specific binders during the three rounds of panning against AXL. Then, we screened the 96 soluble scFv clones from the third round of panning by ELISA and identified 19 scFv clones that bound to AXL-ECD-Fc-6His at pH 5.5. To evaluate the pH-dependent binding of these scFv clones, we performed ELISA at pH 5.5 and 7.4 in parallel. We found that 16 unique scFv clones (E10 and E12 shared the same sequence and G1 and C3 had a mix of sequences) had positive signals (at least 3-fold higher than the background signal of the non-binder E6 and H8 scFv clones) against AXL-ECD-Fc-6His at pH 5.5 (Figure 1a), without any binding to TYRO3-ECD-Fc-6His or to the Fc domain alone at both pHs. Nine clones (indicated with a star) demonstrated a strong binding to AXL-ECD-Fc-6His at pH 5.5 and very low binding at pH 7.4, with a signal at least 5-fold higher at pH 5.5 than at pH 7.4: pH-dependent binding activity ratio (PAR) > 5 (Figure 1b).

We reformatted these 9 scFv clones as fully human IgG1, in which potential N-glycosylation sites were removed before cloning and expression in mammalian cells. All of these antibodies were well produced by transient transfection in HEK293T cells with similar yields after purification using protein A. Lastly, after ELISA to compare their binding to AXL-ECD-Fc-6His at pH 6 and pH 7.4 (Figure S1), we selected the D4 and G10 clones for further characterization (hereafter referred to as D4apH and G10apH). It should be noted that some recombinant divalent IgG clones lost their pH-dependency compared with the monovalent scFv clones, while still binding to AXL.

### 3.2. Characterization of pH-Dependent Anti-AXL IgG Clones

**Figure 2:**
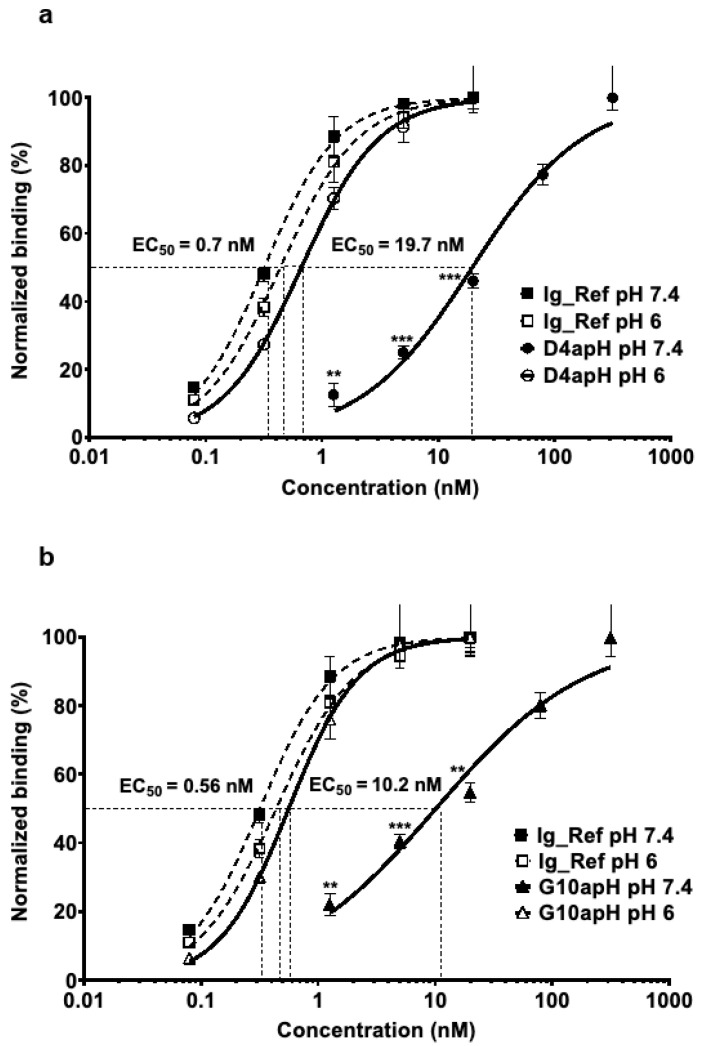
IgGs D4apH and G10apH display lower binding to AXL at a physiological pH than at an acidic pH. The binding activities of (**a**) D4apH and (**b**) G10apH were assessed at increasing concentrations (from 0.08 to320 nM) at acidic (pH 6) and physiological (pH 7.4) pHs by ELISA. Antibody binding was normalized to the mean signal at the highest concentration of each antibody. Error bars show the standard deviation of technical triplicates ** *p* < 0.01, *** *p* < 0.001 (unpaired two-tailed *t*-test).

ELISA using increasing concentrations of D4apH and G10apH confirmed that these two divalent human IgG1 clones bound to AXL-ECD-Fc-6His in a pH-dependent manner. Their EC50 at pH 6 were in the sub-nanomolar range (0.7 nM [0.6–0.8] for D4apH and 0.6 nM [0.5–0.7] for G10apH) and increased by a factor of 28 at pH 7.4 (19.7 nM [15.4–25.2]) and 18 (10.2 nM [7.2–14.3]) (Figure 2a,b). Their binding activity at pH 6 was comparable to that of a non-pH-dependent anti-AXL antibody (Ig_Ref selected from a previous non-pH biased phage selection) at pH 7.4 (EC50 = 0.4 [0.4–0.5] (Figure 2). This result showed that the pH-dependent selection process did not lead to the identification of low-affinity clones.

**Figure 3:**
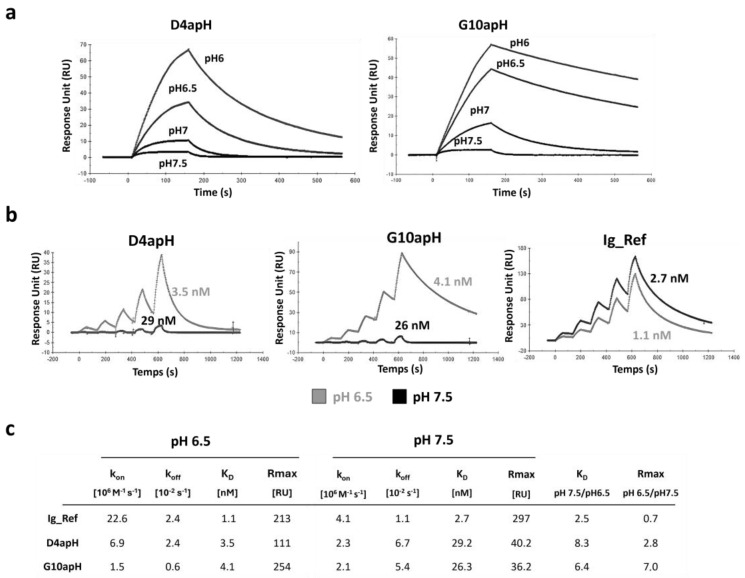
The affinity of the anti-AXL IgGs D4apH and G10apH is lower at a physiological pH than at an acidic pH. (**a**) SPR sensograms of D4apH and G10apH at different pHs. Antibodies were injected at 0.75 nM concentration in PBS at different pHs (pH 6, 6.5, 7, 7.5) on pre-immobilized AXL-ECD-Fc-6His. (**b**) Kinetic titration of the indicated antibodies at pH 6.5 and pH 7.5. The equilibrium dissociation constants were determined by an injection of increasing concentrations of antibodies (five 2-fold increases) at pH 6.5 or pH 7.5. The antibody affinity was determined using a bivalent binding model. (**c**) Summary table of IgG kinetic constants at pH 6.5 and pH 7.5.

Then, we quantified the affinities of the two antibodies at different pHs using SPR. At pH 6.5, both antibodies bound to AXL with similar high affinity (3.5 for D4apH and 4.1 nM for G10apH). However, their binding kinetics were different. G10apH displayed a 4-fold slower dissociation constant than D4apH (0.6 vs. 2.4 10^−3^ s^−1^), whereas D4apH showed a faster association constant than G10apH. Increasing the pH to 7.5 resulted in a decrease in affinity by 8.2- and 6.4-fold for D4apH and G10apH, respectively. It must be noted that the affinity constant of these two antibodies at pH 7.5 could have been overestimated due to the very low maximum response unit obtained at this pH (Figure 3a). This affinity decrease was due to the faster dissociation rate of both antibodies and also a lower association rate for D4apH. The pH effect was explained by an affinity decrease and also by a lower binding capacity, as evidenced by the much lower Rmax at pH 7.5. This may be due to the reduced accessibility of the targeted epitope at a neutral pH. The anti-AXL antibody Ig_Ref (selected using a classical panning at neutral pH) did not show any pH-dependency in this assay (Figure 3b,c).

### 3.3. In Cellulo Binding of pH-Dependent Antibodies

**Figure 4:**
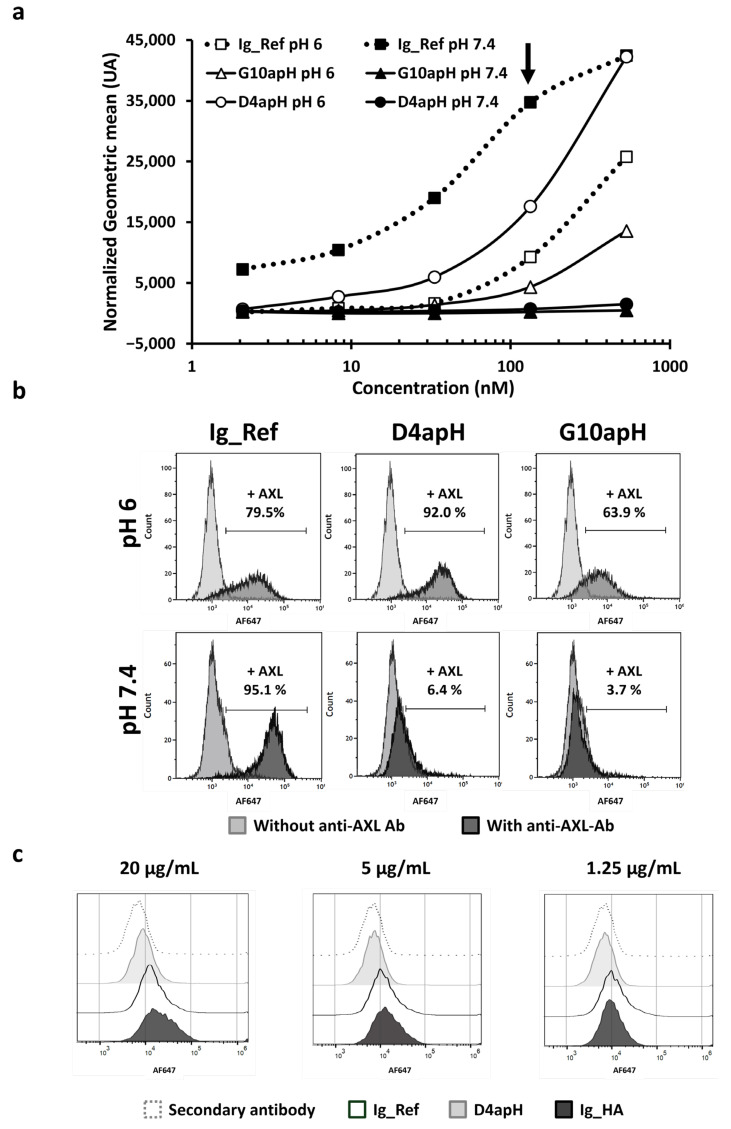
The anti-AXLs G10apH and D4apH display pH-dependent binding to AXL expressed in breast cancer cells and healthy myoblasts. (**a**) Antibody binding with a dose response was assessed by flow cytometry on MDA-MB-231 cells at pH 6.0 and pH 7.4. Normalized geometric mean fluorescent intensity (MFI) signals were determined by subtracting the geometric mean fluorescence intensity of the secondary antibody alone. (**b**) Representative image of antibody binding (at 133 nM from Figure 4a, arrow) by flow cytometry on MDA-MB-231 cells. (**c**) Antibody binding to AXL expressed on immortalized myoblasts AB1167 was evaluated at pH 7.4 by flow cytometry. Ig_HA, high-affinity anti-AXL antibody.

After demonstrating the pH-dependency of D4apH and G10apH in vitro by ELISA and SPR, we sought to confirm their pH-dependent binding on MDA-MB-231 triple-negative breast cancer cells that strongly express AXL [24]. At pH 6, D4apH and G10apH bound to more than 63% of labeled cells (flow cytometry). Despite its faster dissociation constant, D4apH showed a better binding to AXL on MDA-MB-231 cells (Figure 4a,b) and gave a stronger signal than the anti-AXL Ig_Ref antibody. Conversely, at pH 7.4, D4apH and G10apH bound to less than 6% of cancer cells. At this physiological pH, only Ig_Ref bound to more than 95% of cells. As myoblasts also express AXL (confirmed by immunohistochemistry: The human protein atlas; www.proteinatlas.org), we performed flow cytometry of immortalized myoblasts (AB1167) after incubation with the same anti-AXL antibodies at pH 7.4 and found that D4apH did not bind to these cells (Figure 4c; light gray histogram), unlike the non-pH-dependent Ig_Ref antibody (Figure 4c; white histogram) when used at a high concentration. AXL expression on myoblasts was confirmed using high affinity anti-AXL Ig-HA (black histogram). We did not test the G10apH antibody on myoblasts because at pH 6, it showed weaker binding than D4apH on cancer cells. In conclusion, flow cytometry analysis confirmed that the two pH-dependent antibodies, D4apH and G10apH, specifically bound to AXL-expressing breast cancer cells at pH 6 but not to AXL-expressing cancer and healthy cells at a physiological pH.

### 3.4. Epitope Mapping and Mechanism of pH-Dependency

**Figure 5:**
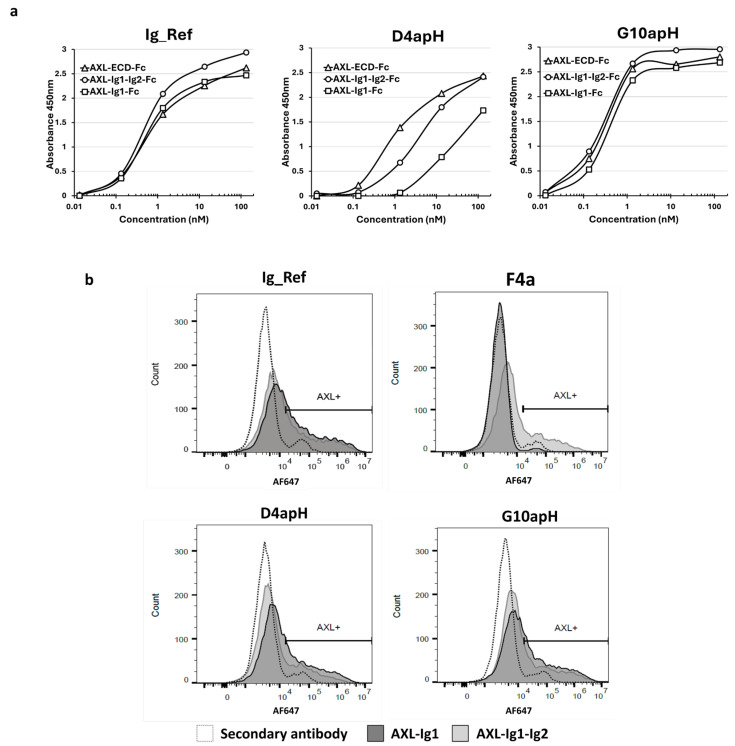
The two best pH-dependent antibodies predominantly bind to epitopes in the Ig-like 1 domain of AXL. (**a**) Antibody binding to the Ig1 and Ig1-Ig2 domains of AXL was evaluated by indirect ELISA. (**b**) Antibody binding to the Ig1 and Ig1-Ig2 domains of AXL was evaluated by flow cytometry in HEK293-Fc-TM cells that transiently express these two domains in a monovalent form. Anti-AXL pH-dependent F4apH antibody binding is specific to the Ig2 domain.

The AXL ECD is constituted by four regions (i.e., two Ig-like domains at the N-terminus, followed by two fibronectin domains) before the transmembrane region [25]. To identify the D4apH and G10apH epitopes, we expressed the first N-terminal Ig domain (Ig1) or both Ig domains (Ig1-Ig2) of human AXL fused to the human IgG1 Fc domain as soluble proteins and used them and the whole AXL-ECD-Fc as antigens in an indirect ELISA. The two pH-dependent antibodies, D4apH and G10apH, and the non-pH-dependent antibody Ig_Ref recognized the three recombinant proteins (Figure 5a). G10apH and Ig_Ref similarly bound to Ig1-Fc, Ig1-Ig2-Fc, and AXL-ECD-Fc, indicating that their epitope is located in the AXL Ig1 domain. D4apH showed weaker binding to Ig1-Fc than to Ig1-Ig2-Fc and AXL-ECD-Fc, indicating that its epitope is mainly located in Ig1 but with the participation of Ig2 either directly or through structural changes. After transient transfection in HEK-293-Fc-TM cells (expressing only the transmembrane Fc), Ig1-Fc and Ig1-Ig2-Fc were monovalently expressed on the surface of HEK293-Fc-TM cells. Flow cytometry analysis showed that D4apH and G10apH similarly bound to Ig1-Fc and Ig1-Ig2-Fc expressed at the surface of HEK293-Fc-TM cells (Figure 5b), confirming that their binding sites are located in Ig1, unlike the F4apH antibody, another pH-dependent antibody derived from this selection (Figure 1), which recognizes the Ig2 domain.

**Figure 6:**
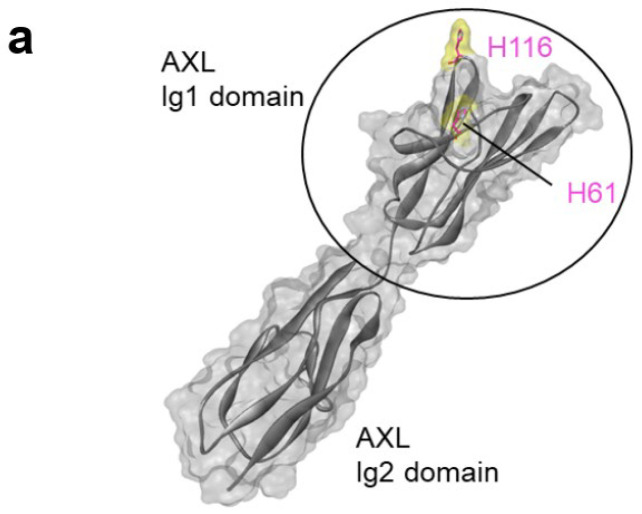

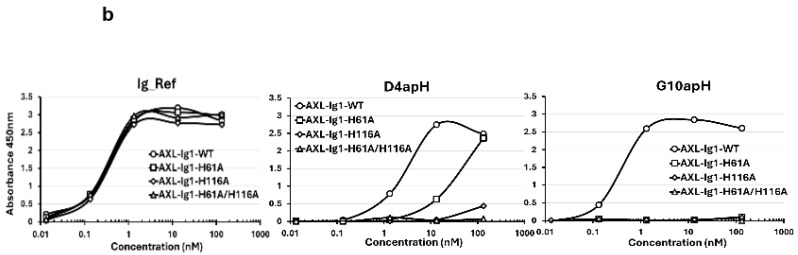
Mutation of a single histidine residue to alanine in the Ig1 domain of AXL leads to the loss of pH-dependent antibody binding. (**a**) Schematic representation of the AXL structure (PDB 2C5D, chain C). The solvent-accessible surface of AXL is in gray. His61 and His116 are shown as pink sticks and their surface is yellow. The docking region on AXL is composed of the Ig1 and Ig2 domains (black circle). (**b**) Antibody binding was evaluated by indirect ELISA using AXL Ig1-Fc or AXL Ig1-Ig2-Fc carrying or not the His61Ala and/or His116Ala mutations, as indicated.

Among the twenty natural amino acids, only histidine contains a side chain with a pKa close to 6–6.5 [26]. We hypothesized that histidine residues could be implicated in the antibody–antigen interaction, thus explaining the pH-dependency. Due to the design of the synthetic HuscI library used in this study, no histidine residue was present in the antibody paratope [16]. We then tested the hypothesis that some of the histidine residues present in AXL Ig1 could participate as D4apH and G10apH binding epitopes. The AXL Ig1 domain contains only two histidine residues that are exposed at the protein surface (positions 61 and 116) (Figure 6a). Therefore, we produced a mutated AXL Ig1 domain in which His61 and/or His116 were mutated to alanine (His61Ala, His116Ala, His61Ala-His116Ala). ELISA (as performed in Figure 5) with these three mutated AXL Ig1-Fc variants (Figure 6b) showed that all mutations affected the binding of D4apH and G10apH but not of the pH-independent Ig_Ref antibody. Moreover, D4apH and G10apH were differently affected by these mutations. D4apH binding was strongly decreased by the His116Ala mutation and to a lesser extent by the His61Ala mutation. Conversely, both mutations completely abolished G10apH binding to AXL. This demonstrated that both histidine residues in the AXL Ig1 domain are part of D4apH and G10apH epitopes and that the two antibodies have overlapping but not identical epitopes.

**Figure 7:**
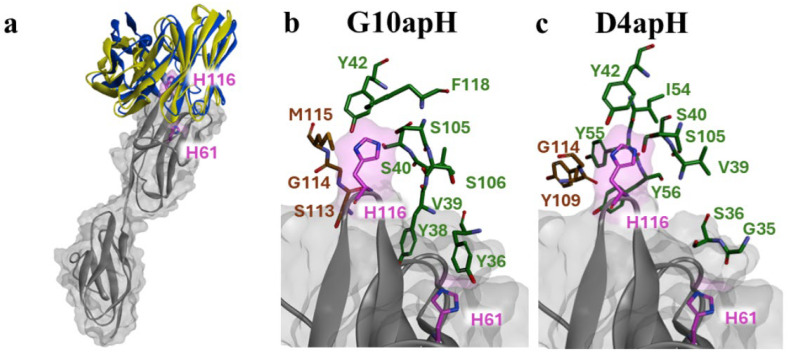
Structural details of the predicted interactions between pH-dependent monoclonal antibodies and Ig-like domains 1 and 2 of AXL. (**a**) Overview of the top poses for G10apH and D4apH. The AXL surface is depicted in grey, with histidine residues highlighted in pink. Atoms are represented as stick models in pink, with oxygen atoms in red and nitrogen atoms in blue. The ribbon representation is blue and yellow for G10apH and D4apH, respectively. (**b**,**c**) Close-up views of the interaction sites on AXL with antibodies, highlighting key residues near His61 and His116 (Sup info, Figure 1b). In the top pose of Cluster 1, the residues of G10apH (**b**) and D4apH (**c**), predicted to be capable of forming π-stacking or hydrogen bond interactions, are shown as stick models. The heavy chain (VH) residues are colored brown while the light chain (VL) residues are green. Oxygen atoms are depicted in red and nitrogen atoms in blue.

The binding modes of each antibody were analyzed using a rigid-body docking approach, followed by the prediction of non-covalent interactions via the RING server (https://ring.biocomputingup.it/accessed on 17 March 2023). The predicted binding modes indicate that pH-dependent antibodies D4apH and G10apH interact with similar contact regions within their Complementarity-Determining Regions (CDRs) to the same region of AXL. In predicted modes of binding, histidine 116 is central to the interaction surface, while histidine 61 is peripheral (Figure 7a). This suggests potential π-stacking interactions with aromatic residues (Tyr, Phe) and hydrogen bonds with hydroxyl-bearing residues (Ser, Tyr). Predicted Van der Waals interactions also contribute to binding stability. The analysis shows that residues from D4apH and G10apH primarily interact with the two histidines through their side chains, with additional hydrogen bonds potentially forming with the protein backbone (e.g., L:54 Ile, L:39 Val, H:114 Gly; see Supplementary Figure S2).

The binding modes and nature of non-covalent interactions provide insights into the mechanism of pH-dependent interactions for both antibodies and elucidate the differences observed between them. At pH 5.5, protonation of histidine enhances hydrogen bond formation, strengthening antibody binding compared to neutral or alkaline conditions. His61 appears less critical for antibody interaction than His116, which is involved in a dense network of interactions. D4apH could form two hydrogen bonds with His61, whereas G10apH might engage through one π-stacking interaction and one hydrogen bond (Figure 7b,c). The aromatic nature of L:Tyr36 in G10apH could play a significant role, as indicated by the pronounced effect of His116 mutation on G10apH binding. His116′s participation in numerous hydrogen bonds makes its protonated state at an acidic pH crucial for the observed pH-dependent effects. Substituting His116 with alanine disrupts this interaction network, significantly decreasing the binding affinity. The proposed binding model underscores the importance of the L:38–42 and L:105–106 regions of the antibodies for pH-dependent interactions.

## 4. Discussion

There are very few tumor-specific antigens targeted by antibodies for therapeutic purposes. Currently, most molecules targeted by therapeutic antibodies are overexpressed in tumors, but they are also expressed at low levels in various healthy tissues. This low expression in healthy tissue leads to toxicity with high-affinity antibodies, such as the skin rashes observed with the cetuximab antibody targeting EGFR. These toxicities are exacerbated when using new immunotherapies, such as ADCs and T-cell engagers. Tumor cells exploit glucose to proliferate, a phenomenon described for the first time by Otto Warburg in 1956 and called the “Warburg effect” [27]. The high consumption of glucose generates an acidic tumor microenvironment through the concomitant release of lactate and protons (H+) by tumor cells [12]. This acidification has been visualized in pre-clinical models and patients using chemical exchange saturation transfer (CEST) magnetic resonance imaging [28,29]. The low pH of the tumor microenvironment could be exploited to increase the antibody specificity and decrease their toxicity to healthy tissues.

Here, we described an easy and rapid method, based on the selection of an scFv-phage library at an acidic rather than a neutral pH, to generate antibodies that can bind to a tumor-specific antigen at an acidic pH without or with very low binding at a physiological pH. We were surprised to rapidly select several different scFv clones with a highly pH-dependent binding capacity without the need to perform time-consuming 3D structure analyses of the antibody/target complex in order to generate pH-dependent antibodies by mutagenesis, as previously described [30,31] or by large mutational scanning of the antibody CDR [32]. Reformatted to IgG, two identified scFv clones showed very good binding to AXL expressed on cancer cells at an acidic pH but not at a physiological pH. Importantly, contrary to a high affinity anti-AXL antibody, these antibodies did not bind to myoblasts that weakly express AXL, used as surrogates of healthy tissues, at pH 7.4. This low binding to normal tissue cells is crucial to decrease the toxicities of future ADCs or T-cell bispecific engagers against AXL.

Using computational docking and point mutations, we demonstrated that the pH-dependent binding capacity of these antibodies is due to the presence of histidine residues in the AXL epitope. Indeed, histidine is the only amino acid that is ionized at pH 6 (slightly acidic) in function of its molecular microenvironment. Based on this observation, we hypothesize that our selection strategy using an acidic buffer could be applied to identify antibodies against other tumor-associated antigens that harbor exposed histidine residues in their extracellular domain. A recent study described the selection of pH-dependent anti-EGFR antibodies [33] using a similar approach: panning at pH 6–6.5 and elution at a neutral pH. Here, we found that the whole selection process can be performed at pH 6 and that elution at pH 7.4 is not necessary. As these anti-EGFR antibodies also targeted histidine residues, this could be a general mechanism of pH-dependent antibodies. Similarly, Johnston et al. [34] demonstrated that VISTA is a pH-dependent ligand due to a patch of histidine residues and that antibodies targeting this patch display pH-dependency. Our strategy is not limited to epitope containing histidine, indeed, in the meantime we have been able to generate pH-dependent antibodies against a small protein that do not contain histidine residues (manuscript in preparation). Despite a relatively low koff, the scFv clones identified in our study could be used to produce CAR-T cells, thus avoiding the toxicity observed when using high-affinity scFv clones [35]. An alternative approach recently developed is to engineer the crystallizable fragment (Fc domain) of an antibody to bind preferentially to Fc receptors on immune effector cells only at an acidic pH [36]. This allows for the easy conversion of any antibody into a tumor-specific one by simply replacing its Fc domain. However, our approach can additionally prevent the antibody from binding at a physiological pH to blood-circulating tumor-released targets, like CEACAM5, CD20, or tyrosine kinase receptor, such as HER2 and EGFR, for example. This is important because such binding reduces the antibody availability and efficacy.

## 5. Patents

T.M., P.M. and B.R. are inventors of patents concerning anti-AXL pH-dependent monoclonal antibodies.

## Data Availability

Sequences of pH-dependent anti-AXL antibodies are available on request due to restrictions (e.g., privacy).

## Supplementary Materials

The following supporting information can be downloaded at: https://www.mdpi.com/article/10.3390/antib14040083/s1, Figure S1: Identification of IgG clones with the highest pH-dependent binding to AXL; Figure S2: Rigid-body docking of D4apH and G10apH.
