# Supplementary material for: Generation Using Phage-Display of pH-Dependent Antibodies Against the Tumor-Associated Antigen AXL
Source: Antibodies (Basel). 2025 Sep 30;14(4):83. doi: 10.3390/antib14040083 (PMC12550914; doi:10.3390/antib14040083)
Supplement: Supplementary file 1 [file antibodies-14-00083-s001.zip › antibodies-3887166-Supplementary Materials.pdf]

1A

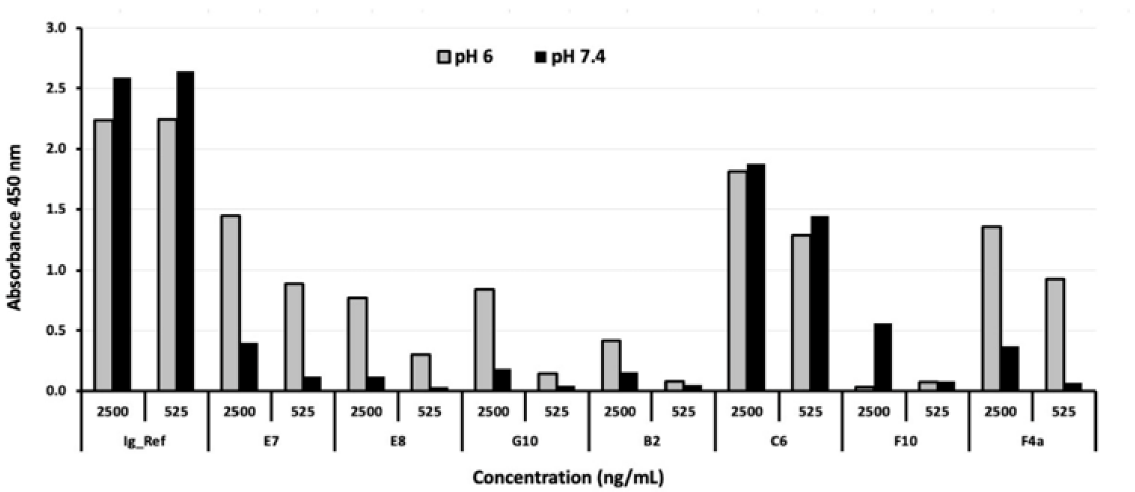

1B

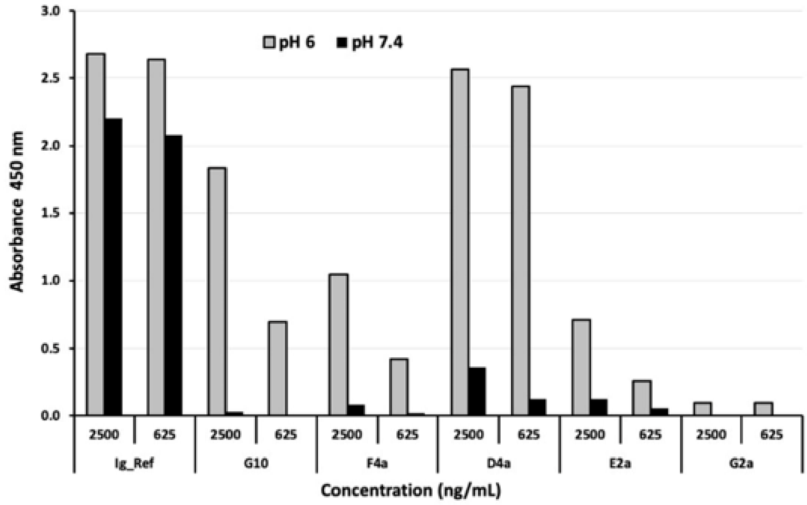

Supplementary Figure S1. Identification of lgG clones with the highest pH-dependent bindingto AXL. Two different ELISA tests (A, B) were performed to identify the most pH-dependent lgGclones. The binding activities ofeach IgG clones were assessed at two concentrations (525or 625 ng/mL and 2500 ng/mL) at acidic (pH 6) or physiological pH (pH 7.4). The Ig Refcontrol antibody displays low pH-dependent binding to AXL.

a

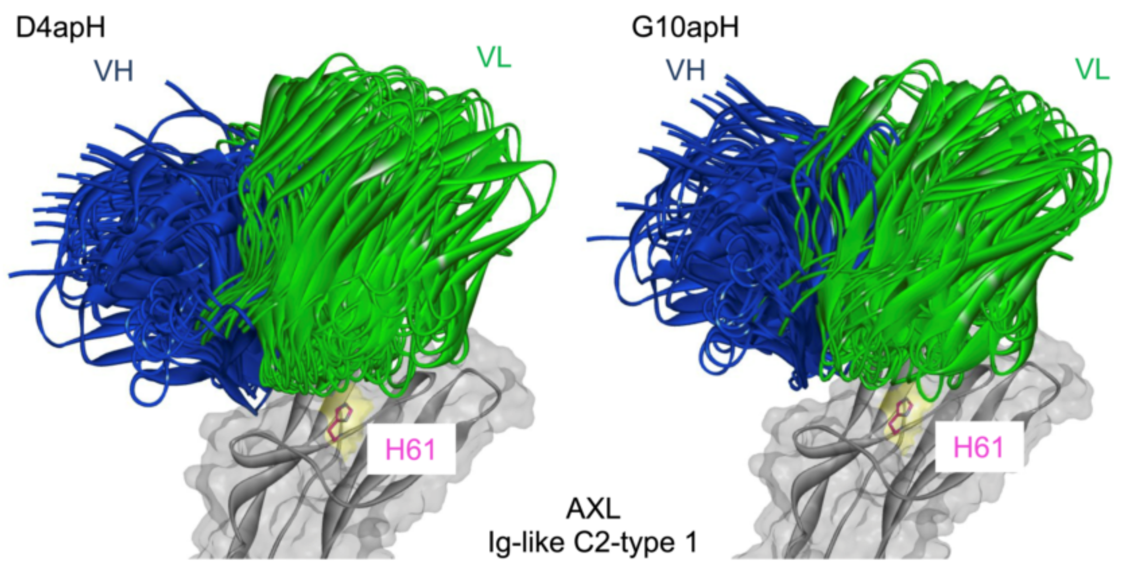

b

D4apH					G10apH				
**Axl His61**	Pi-stacking	Frequency	Hbond	Frequency	**Axl His61**	Pi-stacking	Frequency	Hbond	Frequency
			L:35 Gly	0.47		L:36 Tyr	0.97	L:36 Tyr	0.17
			L:36 Ser	0.37					
**Axl His116**	Pi-stacking	Frequency	Hbond	Frequency	**Axl His116**	Pi-stacking	Frequency	Hbond	Frequency
	L;42 Tyr	0.74	L:39 Val	0.6		L;38 Tyr	0.32	L:38 Tyr	0.67
	L:55 Tyr	0.26	L:40 Ser	0.75		L;42 Tyr	0.51	L:39 Val	0.41
	L;56 Tyr	0.41	L:42 Tyr	0.73		L:118 Phe	0.19	L:40 Tyr	0.23
			L:54 Ile	0.21				L:42 Tyr	0.89
			L:55 Tyr	0.23				L:105 Ser	0.89
			L:105 Ser	0.78				L:106 Ser	0.42
			H:109 Tyr	0.28				H:113 ser	0.23
			H:114 Gly	0.68				H:114 Gly	0.53
								H:115 Met	0.29

Supplementary Figure S2. (a) Docked poses of Cluster 1 for D4apH (left) and G10apH (rightperformed with ZDOCK on the Ig-like C2-type 1 of AXL. The diversity within Cluster 1 isdepicted by 10 out of 91 poses for D4apH and 15 out of 134 poses for G10apH, respectively(AXL surface in grey, VH in blue, VL in green). As shown, the binding mode is similar for bothantibodies in Cluster 1. The His6l of AXL is visible whereas His1 16 is buried by the antibodies. (b) Frequency of putative non-covalent interactions between antibody residues andHis61/His1 16 of AXL. 'L' denotes residues firom the light chain and 'H' from the heavy chainof the antibodies. The frequencies of the identified interactions were calculated based on thenumber of poses within each cluster where the interaction was predicted by RING. Also, vander Waals interactions were not included in this analysis.
